# Microbially Induced Carbonate Precipitation (MICP) as a Sustainable Technology, Mechanism and Application: A Review

**DOI:** 10.3390/ma19194051

**Published:** 2026-09-22

**Authors:** Puchao Jia, Wei Zhang, Wansheng Pei, Gaosen Zhang, Tuo Chen, Guangxiu Liu

**Affiliations:** 1 State Key Laboratory of Ecological Safety and Sustainable Development in Arid Lands, Northwest Institute of Eco-Environment and Resources, Chinese Academy of Sciences, Lanzhou 730000, China; jiapuchao19@mails.ucas.ac.cn (P.J.);; 2 Key Laboratory of Extreme Environmental Microbe of Gansu Province, Northwest Institute of Eco-Environment and Resources, Chinese Academy of Sciences, Lanzhou 730000, China; 3 University of Chinese Academy of Sciences, Beijing 100049, China; 4 State Key Laboratory of Cryospheric Science and Frozen Soil Engineering, Northwest Institute of Eco-Environment and Resources, Chinese Academy of Sciences, Lanzhou 730000, China; 5 Microbial Genetic Resource Center of Extreme Environments of Gansu Province, Northwest Institute of Eco-Environment and Resources, Chinese Academy of Sciences, Lanzhou 730000, China

**Keywords:** MICP, biomineralization, environmental remediation, sustainable technologies

## Abstract

Microbially induced carbonate precipitation (MICP) is a sustainable biomineralization technology with extensive potential in environmental remediation, geotechnical engineering, and materials science, characterized by its eco-friendliness, low energy consumption, and high compatibility. This paper systematically reviews the biochemical mechanisms, critical influencing factors, and recent engineering advancements of MICP. We first explore the metabolic pathways driven by urease and carbonic anhydrase (CA), emphasizing the synergistic effects of microbial strain selection and environmental parameters—such as temperature, pH, and substrate composition—on mineralization efficiency. Furthermore, the review evaluates the latest application cases, including the immobilization of heavy metals and radionuclides, remediation of building materials, soil stabilization, CO_2_ sequestration, and the fabrication of novel biomaterials, highlighting MICP’s role in enhancing engineering performance while mitigating environmental risks. By comparing the cost and sustainability of MICP against traditional chemical methods, this paper identifies current technological bottlenecks—such as reaction efficiency and material brittleness—and proposes future research directions, including synthetic biology for strain optimization and artificial intelligence-assisted design for site-specific applications. Ultimately, this review provides a theoretical framework to guide basic research and promote innovative, scalable deployment of MICP in sustainable technology sectors.

## 1. Introduction

Microbially induced carbonate precipitation (MICP) is a widely used biomineralization technique and is generally classified as biologically induced mineralization. Its main principle involves the microbial secretion of two distinct enzymes, urease and carbonic anhydrase. Urease catalyzes urea hydrolysis, whereas carbonic anhydrase catalyzes CO_2_ hydration; both can promote the generation of carbonate ions, which then react with metal ions in the environment to form carbonate precipitates. As early as 1973, research confirmed that soil bacteria (e.g., *Bacillus*) are generally capable of producing calcite crystals [1]. Lowenstam introduced the term “biomineralization” to distinguish between biologically controlled and biologically induced mineralization [2]. Biologically controlled mineralization refers to processes in which organisms exert strict genetic and cellular control over mineral nucleation and growth, typically through specialized cells or organic macromolecules, resulting in minerals with well-defined crystallographic orientation and composition. In contrast, biologically induced mineralization occurs when metabolic activities of organisms modify the local microenvironment (e.g., pH, redox conditions, or ion concentration), leading to mineral precipitation that is not directly regulated by the organism, often producing crystals with heterogeneous composition and arbitrary orientation. MICP belongs to this latter category: carbonate precipitation is driven by microbial metabolic activities and changes in the local microenvironment, rather than by genetically controlled cellular processes. Therefore, the term “biomineralization” is introduced here not as general background but to define the mechanistic category of MICP and to provide the conceptual framework for the mechanisms, influencing factors, and applications discussed in this review. Since 2000, with the integration of multiple disciplines, the technology of microbially induced carbonate precipitation has achieved significant applications in a number of fields, such as pollution control of heavy metals and radioactive elements [3,4,5,6,7], remediation and protection of building materials [8,9,10,11], soil stabilization and desert management [12,13,14], CO_2_ sequestration and carbon capture [15,16], and biomaterials [17], among others.

MICP is a complex and delicate process involving multiple biochemical reactions. These primarily include: urea decomposition, amino acid ammonification, denitrification, reduction by heterotrophic sulfate-reducing bacteria, photosynthesis, methane oxidation, and other metabolic processes. Many studies have shown that the crystalline nature of microbially precipitated carbonate polymorphs (e.g., calcite, dolomite, vaterite, hydroxyapatite, and aragonite) is highly dependent on environmental conditions and not just on microbes or enzymes [7,17,18,19,20]. The key factor in improving the efficiency of microbial precipitation of carbonates is the combination of organisms and environmental conditions, including (1) microbial strain differences, (2) temperature, (3) pH, and (4) substrate.

Although MICP has been widely studied, the literature remains fragmented across disciplines. Studies on different pathways, influencing factors, and applications are often reported separately [21], and experimental conditions vary greatly, making results difficult to compare [22]. Moreover, most studies remain at the laboratory stage, while field demonstrations and commercial applications are still limited, and environmental risks such as ammonium release and increased salinity are often overlooked. A critical synthesis is therefore needed to clarify which factors are most influential, under what conditions optimal performance can be achieved, and which pathway is most suitable for each application. This review addresses these issues by examining the biochemical mechanisms, influencing factors, and engineering applications of MICP.

## 2. Microbially Induced Carbonate Precipitation Pathways and Mechanisms

### 2.1. Urease

Although several processes such as photosynthesis, ammonification, and denitrification have been related to and studied for their ability to precipitate carbonate polymorphs, urea decomposition is the most commonly used technique for calcium carbonate precipitation. In the MICP process, urease is a key enzyme that catalyzes the hydrolysis of urea into ammonia and CO_2_. This reaction increases the local pH, thereby promoting calcium carbonate precipitation [21]. More specifically, urea hydrolysis produces ammonia and carbamate, and carbamate subsequently hydrolyzes spontaneously to form a second molecule of ammonia and carbonic acid. The resulting ammonia species increase the local pH, while carbonic acid equilibrates with bicarbonate and carbonate ions, creating favorable conditions for carbonate precipitation in the presence of available metal cations.

In engineering applications, the efficiency of the urease pathway is affected by a number of factors, including the type of microorganism, the concentration of urea and calcium sources, and the environmental pH. It has been shown that appropriate cell and urea concentrations can significantly increase the efficiency of MICP and affect the morphology and mineralogical properties of calcium carbonate crystals [22]. In addition, the urease pathway can be used in combination with other biochemical pathways to improve the overall effectiveness of MICP [23]. The urease pathway is also attractive because it can produce a rapid and substantial increase in pH and carbonate alkalinity, which favors the supersaturation required for calcium carbonate nucleation [21]. However, excessive urea hydrolysis may lead to rapid precipitation and heterogeneous crystal formation [22], and the accumulation of ammonium by-products may also influence the local environment. Therefore, balancing urease activity, substrate supply, and environmental conditions is important for optimizing the urease-driven MICP process. In conclusion, the role of urease in MICP engineering applications is crucial, and with the in-depth study of the MICP mechanism, the application of the urease pathway will be more promising. Figure 1a provides an illustration of the urease process.

Ureolysis yields two moles of ammonium per mole of urea hydrolyzed, and the resulting ammonium may be subject to volatilization as ammonia, nitrification followed by nitrate leaching, or transport to receiving waters, where it can contribute to eutrophication if the treated site is not adequately managed [20,21]. The concomitant rise in pH and salinity may further perturb indigenous microbial communities and exert phytotoxic effects on vegetation [18,21]. In addition, the accumulation of ammonium salts poses a risk of corrosion to concrete and steel in built environments [8,9]. These environmental consequences are frequently overlooked in laboratory investigations, which tend to prioritize precipitation efficiency over the fate of reaction by-products [20]. For field-scale applications, ammonium recovery, nitrogen management, or the adoption of non-ureolytic pathways should therefore be considered [16]. This consideration is particularly pertinent to large-volume soil treatment, where the total ammonium load may be substantial.

### 2.2. Carbonic Anhydrase

Compared to urease, carbonic anhydrase (CA) also plays an important role in the MICP process and can catalyze the rapid interconversion of CO_2_ with water, thus promoting the precipitation of carbonate [24]. During the MICP process, CA increases the concentration of HCO_3_^−^ and CO_3_^2−^ by accelerating the hydration reaction of CO_2_, thereby increasing the supersaturation of carbonate and promoting the formation of CaCO_3_ [25]. Unlike urease, which primarily depends on urea hydrolysis, CA directly targets the CO_2_–bicarbonate–carbonate equilibrium, making it particularly relevant to carbon capture and CO_2_ sequestration scenarios [15,16].

Studies have shown that CA is not only effective under laboratory conditions, but also exhibits significant effects in practical applications [21,26,27,28,29]. The CA pathway can function under conditions where urea is limited or where CO_2_ is abundant, and it may therefore complement urease-driven MICP in mixed or engineered systems [28,29]. In addition, CA activity can be affected by environmental factors such as pH, temperature, and the availability of metal ions, which influence both enzyme stability and the carbonate equilibrium. In conclusion, CA plays a key role in the MICP process and shows great promise in practical applications, providing innovative solutions for soil stabilization, heavy metal fixation and carbon capture. Figure 1b provides an illustration of the CA process.

### 2.3. Other Metabolic Pathways Involved in Carbonate Precipitation

Although urease and CA are the most extensively studied pathways, carbonate precipitation can also be induced by ammonification, denitrification, sulfate reduction, photosynthesis, and methane oxidation [18,30]. These pathways differ in their carbon and nitrogen sources, energy requirements, and environmental by-products, and they are therefore not interchangeable. These pathways should not be treated as equivalent alternatives. Ureolysis is the fastest and most widely used, but it produces ammonia; denitrification and sulfate reduction avoid ammonia but require anoxic conditions; photosynthesis is limited by light; and methane oxidation is slow [26,30]. The choice of pathway should therefore be matched to the target application, environmental conditions, and acceptable by-products.

### 2.4. Mineral Products and Characterization of MICP

Based on the metabolic pathways discussed above, MICP ultimately leads to the precipitation of carbonate minerals, predominantly calcium carbonate (CaCO_3_). The morphology and polymorph of the precipitated CaCO_3_ are key indicators of the mineralization process and are strongly influenced by the local microenvironment. Figure 2 shows representative SEM images and an XRD pattern of the calcium carbonate formed after MICP. The SEM images at different magnifications (scale bars: 1 μm, 5 μm, and 100 μm) reveal rhombohedral calcite crystals, scattered spherical particles, and a dense fine-grained coating on sand surfaces. The XRD pattern confirms calcite as the dominant mineral phase, with quartz peaks originating from the sand substrate.

## 3. Factors Affecting the Efficiency of MICP

### 3.1. Microbial Strain Differences

MICP is quite common among microorganisms, many of which have urease activity and under the right conditions produce urease enzymes that hydrolyze urea to produce carbonate ions [30]. At the same time, researchers conducted studies on the ability of ureolytic bacteria to induce carbonate precipitation, and different types of strains were able to produce different amounts of urease and calcium carbonate precipitation [31,32,33,34,35,36,37,38]. It has been shown that mixed cultures containing ureolytic and non-ureolytic bacterial strains can significantly increase the level of calcium carbonate precipitation [39]. The use of such mixed cultures not only improves the precipitation efficiency, but also allows for effective operation at high temperatures and alkaline environments, which is very favorable for MICP applications. In addition, different strains showed different adaptations under different environmental conditions. For example, *Sporosarcina* sp. HYO08 and *Bacillus* sp. JH7 strains exhibit greater alkali and salt tolerance, respectively [40]. This difference in physiological properties makes these strains uniquely advantageous in different application scenarios. In summary, the selection of suitable strain species is the key to improving the precipitation efficiency of calcium carbonate. In addition, studies have shown that cooperation between different strains can enhance the MICP process [41,42]. These studies provide an important theoretical basis and practical guidance for future applications in the fields of construction, soil reinforcement and environment.

Although numerous strains have been reported to induce carbonate precipitation, the reported efficiencies are not directly comparable because of differences in cell density, urea and calcium concentrations, pH, temperature, and medium composition [22,31,43]. Among the factors reviewed here, microbial strain selection and substrate composition appear to be the most influential [31,37,39], whereas temperature and pH mainly modulate reaction rates and crystal polymorphs [43]. Optimal conditions are therefore system-specific rather than universal: ureolytic MICP generally performs best at pH 7.5–9.0 and 25–35 °C, while CA-driven systems may tolerate lower urea concentrations and higher CO_2_ availability [24,25,28,43]. The transferability of results between soil types remains uncertain, because grain size, mineralogy, organic matter, and pore structure all affect transport, retention, and cementation uniformity. For each application, the most promising pathway differs: urease-based MICP is preferable for rapid strength gain and heavy metal immobilization, whereas CA-based or non-ureolytic pathways are more suitable for CO_2_ sequestration and low-ammonia environments [21,28,29,33].

### 3.2. Temperature

During the MICP process, changes in temperature significantly affect the bacterial urease activity, which in turn affects the precipitation efficiency of calcium carbonate. It was shown that temperature not only affects the precipitation rate of calcium carbonate, but also its final precipitation amount and crystal structure [43]. The experimental results showed that the urease activity of bacteria was higher at higher temperatures during the initial 20 h, but with time, the urease activity decreased more rapidly at higher temperatures than at lower temperatures [19,44]. In addition, temperature has a significant effect on the early nucleation process of calcium carbonate. Through molecular dynamics simulations, it was found that higher temperatures increase the polymerization rate and nucleation activation energy of calcium carbonate, which implies that the precipitation process of calcium carbonate is more rapid under high-temperature conditions [45]. However, an increase in temperature may also lead to a decrease in precipitation efficiency, which is particularly noticeable under high salinity and high temperature conditions [45]. Overall, the effect of temperature on the precipitation efficiency of calcium carbonate is multifaceted, involving the combined effects of several factors such as chemical reaction kinetics, microbial activity, and environmental conditions [37,43,44,45]. Therefore, in practical application, the temperature needs to be reasonably adjusted to optimize the precipitation efficiency of calcium carbonate according to specific environmental conditions and target requirements.

### 3.3. pH

The pH plays a key role in the carbonate precipitation process. Higher pH promotes the formation of CO_3_^2−^, affecting precipitation efficiency and crystal morphology. Studies have shown that higher pH contributes to the carbonate precipitation. For example, during microbial CA-induced carbonate precipitation, the Ca^2+^ were completely deposited within 48 h at an initial pH of 8.0, 21 h, 15 h and 14 h earlier than at pH 6.0, 6.5 and 7.0, respectively, which suggests that higher pH is favorable for the carbonate precipitation within the pH range of the experiment [46]. Furthermore, it was found that an increase in pH leads to an increase in precipitation efficiency during enzyme-induced MICP, especially at high salt concentrations [47]. In another study, pH and aeration were identified as the two main factors affecting bacterial carbonate precipitation. The results of the study showed a 6.3-fold increase in carbonate precipitation with an increase in pH to 12 [48]. In addition, pH affects the morphology and mineralogical properties of calcium carbonate crystals. In some cases, higher pH leads to a transition of crystals from aragonite phase to calcite phase [49]. It has been shown that pH also has an effect on the microstructure of calcium carbonate precipitates, with changes in pH affecting the composition of particles and crystal morphology [50]. In summary, pH is a key factor affecting the precipitation efficiency and crystal properties of calcium carbonate, which has shown its importance in different experiments and applications.

### 3.4. Substrate

The type, concentration and ratio of substrate are some of the important factors affecting the kinetics and final yield of MICP reaction. Studies have shown that different substrate types can significantly affect the growth of microorganisms and the carbonate precipitation process [51,52]. For example, certain studies have found that the use of different calcium sources (e.g., CaCl_2_, Ca(CH_3_COO)_2_, Ca(C_6_H_11_O_7_)_2_) affects the effect of Cr mineralization during the MICP process, with CaCl_2_ being considered the most effective calcium source [7]. In addition, changes in substrate concentration can significantly affect the efficiency and product properties of MICP. Studies have shown that the initial bacterial cell concentration has a significant effect on the kinetics of urea hydrolysis and carbonate precipitation, and changes in cell concentration lead to changes in the morphology and mineralogical properties of calcium carbonate crystals [22]. In another study, by adjusting the ratio of bacteria to calcium, researchers found that they could modulate the morphology of CaCO_3_ crystals and achieve a high-purity output of calcite-type CaCO_3_ [53]. Optimization of substrate ratios is likewise key to improving MICP efficiency. Studies have shown that the fixation efficiency of heavy metals can be significantly improved by optimizing the concentration ratio of urea to heavy metal ions. For example, in one study, efficient immobilization of Cd^2+^ was successfully achieved by a two-step MICP method, and a high removal rate was maintained even in a high-concentration Cd^2+^ environment [54]. Studies have shown that the addition of MgO greatly improves the efficiency of carbonate precipitation [55,56]. In addition, in research on coastal erosion protection, researchers have also found that the addition of fibers can enhance the durability of bio-cemented sand in the MICP process [57]. In summary, the efficiency and product quality of MICP can be improved by rationally regulating factors such as the type, concentration and ratio of the substrate, providing theoretical support and practical guidance for its subsequent application [58,59].

## 4. Application of MICP Technology System

### 4.1. Heavy Metal and Radioactive Element Pollution Control

Heavy metals usually refer to metallic elements with densities greater than 4.5 g/cm^3^, such as lead (Pb), cadmium (Cd), chromium (Cr), etc., while radioactive elements have the property of spontaneously decaying and emitting rays, such as uranium (U), thorium (Th), radium (Ra), etc. [60]. The chemical nature of heavy metals makes them potentially hazardous in the environment, posing a threat to the surrounding ecosystem and human health if their levels exceed soil environmental quality standards [27], and the enrichment of heavy metals in organisms increases with the level of the food chain [61,62]. The MICP method, on the other hand, demonstrates the advantages of high efficiency, cost-effectiveness and eco-friendliness in the removal of heavy metal contamination. MICP has been reported to have great potential for remediation of heavy metals and radionuclides in the environment [33,63,64,65,66,67].

However, the toxicity of heavy metals affects the growth of microorganisms and the efficiency of MICP; researchers need to improve the efficiency of the MICP process by isolating metal-tolerant microorganisms with urea catabolism ability. In a study, *Lysinibacillus* sp. GY3 was found to be a promising catalyst for the immobilization of heavy metals through the MICP process. The strain exhibited stable urease activity under metal stress, especially in the presence of 1 g/L of Pb^2+^ and Zn^2+^, and was able to maintain more than 70% of the activity [68]. Studies have also shown that the synergistic effect of microbial communities can further improve the removal efficiency of heavy metals. For example, it was found that a microbial consortium of phosphate-solubilizing and urease-producing bacteria was able to significantly increase the stabilization rate of heavy metals, which reached 98.35%, 99.78%, 99.09%, and 92.26% for Cu, Zn, Cd, and Pb, respectively [69]. In addition, MICP treatment using waste leachate-derived urease bacterial consortium can effectively remove cadmium and nickel while improving soil stability [70]. Table 1 summarizes research on MICP for entrapping or removing heavy metals, and Figure 3a provides an illustration of the process.

During the MICP process, the formed calcite can bind heavy metals (e.g., Pb^2+^) to the surface of appropriate divalent cations (e.g., Ca^2+^), and then Pb^2+^ replaces the appropriate divalent cation (Ca^2+^) in the calcite lattice by substitution reaction. In this way, heavy metal ions are transformed from soluble to insoluble forms, i.e., detoxifying heavy metals.

### 4.2. Restoration and Conservation of Crack, Fracture and Weathered Rock

Concrete cracks reduce its service life and performance, and MICP technology provides a new way for crack repair. In practice, crack repair can be realized by introducing calcium carbonate-producing bacteria and related nutrients into concrete cracks. For example, in a field study of cracks in the Fayette sandstone formation at a depth of 340.8 m below ground surface, after 24 injections of urea/calcium solution and 6 injections of microbial (*Sporosarcina pasteurii*) suspension, the injectivity of the cracks was reduced and the pressure drop in the wells was reduced, suggesting that the MICP can effectively seal the cracks [112]. In a laboratory study, the screened *Bacillus cereus* strain with high urease activity was able to achieve more than 90% fracture repair with the addition of 10 g/L sodium carboxymethyl cellulose (CMC-Na) [113]. Table 2 summarizes research on MICP for restoring and conserving building materials, and Figure 3b provides an illustration of the process.

Although laboratory studies have reported high repair ratios, field-scale applications remain relatively few [112,128,131]. This discrepancy can be attributed primarily to the heterogeneity of field conditions and the transport limitations of bacteria and substrates within actual cracks and porous media [51,127,131]. Unlike idealized laboratory specimens, field cracks vary considerably in width, geometry, and connectivity, which complicates the uniform delivery and retention of the treatment solution. In addition, injection strategy and nutrient availability are difficult to control under field conditions, and their spatial and temporal variability can further reduce repair efficiency. Consequently, the gap between laboratory performance and field implementation, rather than a lack of technical feasibility, is the main reason for the limited number of documented field applications.

As an important part of architectural and cultural heritage, stone is often exposed to erosion by weathering and other environmental factors. In order to effectively protect stone, MICP technology has been widely studied and applied in recent years. MICP technology forms a protective carbonate layer on the surface of stone through the biomineralization of specific bacteria, which improves the durability and resistance to weathering of stone [21]. In addition, MICP technology can be used in combination with nanotechnology to enhance the protection of stone surfaces. Studies have shown that silica nanomaterials, as an effective stone reinforcement, can significantly improve the mechanical strength and durability of stone [138]. The application of this nanomaterial provides new ideas and methods for stone protection. In addition, MICP technology can be used in combination with self-healing coating technology to improve the weathering and self-healing capabilities of stone surfaces. By forming a nanostructured self-healing coating on the stone surface, it can effectively resist various erosion factors in the natural environment and extend the service life of the stone [139]. In summary, MICP technology shows a wide range of application prospects in stone protection, and through the combined use with other technologies, it can realize the comprehensive protection and restoration of stone, providing strong support for the long-term preservation of stone.

### 4.3. Soil Reinforcement and Desert Management

In foundation treatment, the microbial mineralization process provides a new way to solve the problems of foundation stability and durability. This technology induces calcium carbonate precipitation under suitable conditions by introducing specific microorganisms into foundation soil, thus filling soil pores, enhancing cementation between soil particles, and improving the mechanical properties and stability of soil. Studies have shown that MICP technology can not only effectively improve the compressive strength of the soil, but also significantly reduce the permeability of the soil, thus improving the overall stability of the foundation [140]. In specific applications, MICP technology produces calcium carbonate precipitates through the metabolic activities of microorganisms, and these precipitates can form bridges between soil particles and enhance the structural strength of the soil. Experimental studies have shown that the precipitation efficiency of calcium carbonate can be significantly improved by optimizing the growth conditions and reaction environment of microorganisms, thus further improving the mechanical properties of soil [70]. Table 3 summarizes research on MICP for soil reinforcement and desert management, and Figure 3c provides an illustration of the process.

The effectiveness of MICP for soil reinforcement depends strongly on soil type, grain size, and cementation solution composition [51,58,127]. Results from homogeneous sand cannot be directly transferred to loess, clay, or saline soils [149,153,157]. In addition, MICP technology can be used to improve the stability of desert soil and enhance its resistance to wind erosion, thus playing an important role in ecological restoration and desertification management. Studies have shown that desert soils treated by Microbial Induced MICP technology exhibit significant structural stability and erosion resistance in wind erosion tests. MICP technique also achieved significant results in enhancing the penetration strength and wind erosion resistance of desert soils, and scanning electron microscopy analysis confirmed the formation of calcium bridges between soil particles, which enhanced inter-particle bonding [150]. In another study applying MICP technology to desert regions, researchers found that the treated soil crust was 12.5 mm thick, contained 0.57% CaCO_3_, and remained intact after being exposed to wind speeds of 30 m/s for two minutes [162]. In addition, MICP technology has been shown to have significant effects in increasing the compressive strength of soil and reducing soil porosity, thus effectively preventing soil erosion [163].

MICP technology not only performs well under laboratory conditions, but also shows great potential in practical applications. In conclusion, MICP technology has significant advantages in improving the stability and wind erosion resistance of desert soils, providing an effective solution for desertification management and ecological restoration. The application of this technology not only helps to reduce soil erosion, but also improves the mechanical properties and environmental adaptability of the soil, providing a broad prospect for future ecological engineering applications [164].

**Figure 3:**
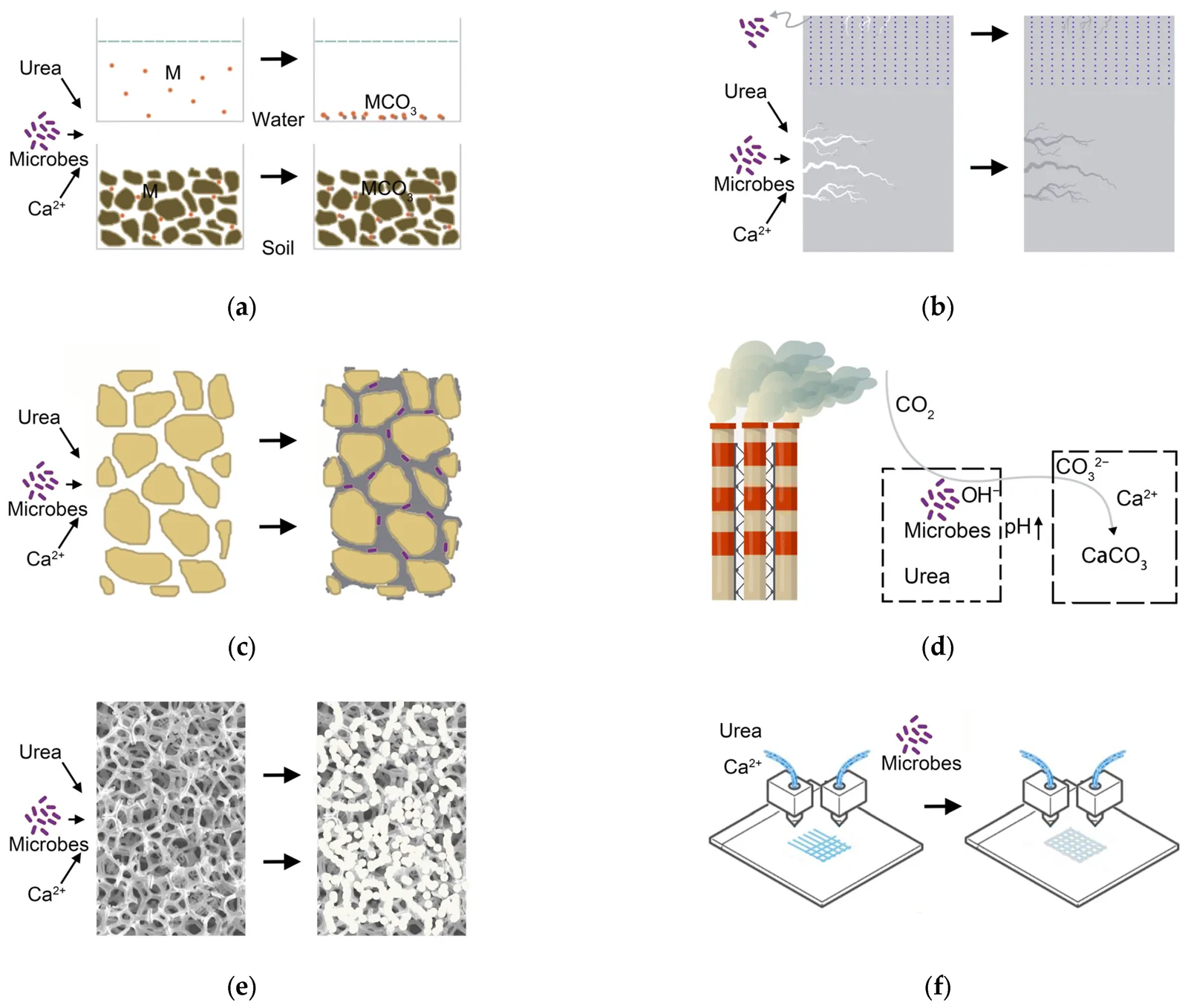
(**a**) Precipitation of metal cations; (**b**) Crack repair; (**c**) Sand fixation; (**d**) CO_2_ capture; (**e**) Bio-brick; (**f**) 3D-Printing through MICP.

### 4.4. CO_2_ Sequestration and Carbon Capture

Strategies for the application of microorganisms in carbon capture technologies aim to fully utilize their unique metabolic capabilities for efficient CO_2_ capture and conversion. For example, some bacteria are capable of producing CA, an enzyme that catalyzes the reaction of CO_2_ with water to form bicarbonate ions, thus facilitating CO_2_ fixation. By introducing the gene encoding CA into suitable microorganisms by means of genetic engineering, engineered strains with efficient CO_2_ capture can be constructed. In one study, the CA gene from *Bacillus megaterium* was introduced into *Bacillus subtilis*, and the constructed recombinant strain showed high efficiency in CO_2_ capture experiments and was able to significantly reduce the CO_2_ concentration in the environment [29]. Researchers further investigated the factors influencing the rate and concentration of carbon dioxide sequestration. The study found that biological factors are strain types, while non-biological factors are primarily pH and medium composition, and that an increase in pH enhances the system’s ability to capture carbon dioxide [165]. Additionally, through Plackett-Burman experiments, it was discovered that the most important medium component involved in carbon dioxide capture is urea, and based on the results, the medium composition was optimized, resulting in a 1.5-fold increase in carbon dioxide absorption by the MICP capture system [165]. Table 4 summarizes research on MICP for CO_2_ sequestration and carbon capture, and Figure 3d provides an illustration of the process.

Microbially induced calcium carbonate precipitation has a broad application prospect in the field of industrial carbon capture, which provides an innovative way to reduce CO_2_ emissions in industrial production processes [166]. In some industrial exhaust emission scenarios, such as cement factories and power plants, microbially induced calcium carbonate precipitation technology can be utilized to capture CO_2_ from exhaust gases. By contacting a solution containing specific microorganisms and calcium ions with the exhaust gas, carbonate ions produced by microbial metabolism react with CO_2_ to form calcium carbonate precipitation, which leads to CO_2_ immobilization. Studies have shown that treatment with microorganisms such as *Sporosarcina pasteurii* in a simulated industrial exhaust gas treatment system can effectively reduce the concentration of CO_2_ in the exhaust gas and achieve a certain degree of carbon capture [169]. The efficiency of CO_2_ capture depends on pH, medium composition, and strain type, with urea being the most important medium component [28,29,165]. However, most studies are still at laboratory scale, and the long-term fate of captured carbon is not well understood.

### 4.5. New Biomaterials and Technologies

The application history of MICP in the field of new building materials has gone through a gradual development process from theoretical exploration to practical application. In the early stage, the research mainly focused on the basic principles of microbial-induced calcium carbonate precipitation and the feasibility verification under laboratory conditions. With the increasing demand for sustainable building materials, MICP technology has gradually gained attention.

MICP technology, as an emerging biomineralization technology, has shown great potential in the preparation of biocement in recent years. This technology can be used not only for soil stabilization but also for the production of environmentally friendly and durable building materials [21,170]. In the application of bio-cement, MICP technology is considered a green building solution because of its ability to reduce carbon emissions, thus slowing down the rate of global warming [171]. Studies have shown that MICP technology has significant results in repairing cracks and surface treatment, which makes it an emerging trend in construction [171]. In terms of durability of microbial cements, studies have shown that MICP-treated samples reinforced by fibers (e.g., jute) have improved long-term performance under simulated real site conditions. This reinforcement method can improve the durability of MICP-treated samples, especially in coastal erosion protection [57]. In addition, the incorporation of fibers improves the mechanical and physical properties of MICP-treated samples [57]. Table 5 summarizes research on MICP for new biomaterials and technologies, and Figure 3e,f provides an illustration of the process.

MICP technology shows significant potential for the preparation of biobricks. With MICP, biobricks can be “grown” using urea from urine as a reaction substrate, combined with biomineralization by microorganisms. This process does not require traditional high-temperature firing, but rather the precipitation of minerals through microbial activity [17]. Specifically, the researchers used the bacterium *Sporosarcina pasteurii* to facilitate the MICP process. This bacterium is able to break down urea in urine to produce carbonate ions, which then combine with calcium ions in the urine solution to form calcium carbonate. This calcium carbonate acts as a bio-cement that binds loose sand particles together to form the shape of bricks. Experimental results showed that the compressive strength of the prepared biobricks could reach 2.7 MPa, which is comparable to that of bricks made by the conventional brick-making process [17]. In addition, another study also investigated the preparation of biobricks using synthetic urine and *Lysinibacillus fusiformis* bacteria. The study showed that the biobricks prepared by this method were 59% higher than the control in terms of compressive strength, while the porosity was reduced to 13% from 22% in the control. This improved strength and reduced porosity are mainly attributed to the minerals formed in the biobricks such as guano, apatite and calcite [180].

MICP technology also faces some challenges in practical applications, such as reaction efficiency, material brittleness, and environmental adaptability. Therefore, combining MICP technology with other advanced technologies has become an important direction to solve these problems. For example, it has been shown that combining MICP technology with plant straw can significantly improve the shear strength and internal friction angle of loess. This combination takes advantage of the natural fiber structure of plant straw and enhances the overall stability of the soil [147]. In addition, the combination of MICP technology with activated carbon can improve the compressibility and water retention of saline soils. The addition of activated carbon not only enhanced the MICP reaction, but also further reduced the compressibility of the soil and improved its water retention capacity [149]. In heavy metal passivation, the combination of MICP technology with inorganic additives has also shown good results. For example, by combining with calcium oxide, MICP technology can effectively passivate heavy metal ions in copper-nickel tailings, which significantly improves the strength and compactness of the tailings [85]. This synergistic effect not only improves the application range of MICP technology, but also provides a low-cost solution for tailings curing.

### 4.6. Technology Readiness and Scalability

The applications reviewed above are at very different stages of maturity, and to avoid overstating the current status of MICP, they can be classified according to technology readiness level (TRL). Most heavy metal immobilization studies [3,4,5,6,7,33,54,64,82], bio-bricks [17,180], bio-tiles [177,178], 3D printing [170,173,175,179], and dental composites [172] remain at the laboratory proof-of-concept stage (TRL 1–3), where feasibility has been demonstrated but field-scale testing has not yet been performed. In contrast, fractured rock sealing [112,128,130,131,133], wellbore leakage mitigation [169], desert soil wind erosion control [162,163], and some slope protection trials [161] have reached the field demonstration stage (TRL 4–6), showing that MICP can be delivered under real field conditions, although long-term performance and cost remain uncertain [112,128,162]. Commercial deployment (TRL 7–9) is currently limited, and no large-scale commercial MICP process is yet established for soil improvement, although biocementation is being piloted by several companies [176]. Key barriers to scaling up include the uniform delivery of bacteria and substrates in heterogeneous soils, ammonium by-product management, the cost of substrates and nutrients, long-term durability under wetting–drying and freeze–thaw cycles, and regulatory acceptance. Future work should therefore report field-scale performance, life-cycle cost, and environmental impact rather than only laboratory precipitation efficiency.

## 5. Limitations and Risks

Despite its potential, MICP is subject to several limitations and risks that warrant careful consideration prior to large-scale implementation. A primary concern is the heterogeneity of precipitation, particularly in coarse-grained or stratified soils, which can result in non-uniform cementation and localized mechanical failure [51,58,127]. Ureolytic MICP additionally generates ammonium and elevates salinity, raising concerns regarding groundwater contamination and vegetation stress [48,61,68]. The long-term durability of bio-cemented materials also remains insufficiently documented, especially under cyclic wetting–drying, freeze–thaw, and chemical attack [57,152,161]. Furthermore, bio-cemented sand and bio-bricks exhibit inherent brittleness and may fail under tensile or shear loading unless reinforced with fibers or additives. Potential ecological impacts include competition between introduced and indigenous microbial communities, as well as alterations in soil chemistry following large-scale substrate injection. Economic and logistical constraints further hinder deployment, given the relatively high costs of substrates, nutrients, and delivery, together with the absence of an established supply chain for field-scale application. In addition, regulatory uncertainty persists because the release of engineered or non-native microorganisms is governed by biosafety regulations that differ across jurisdictions. These risks should be systematically quantified through standardized leaching, ecotoxicity, and durability assessments and reported alongside precipitation efficiency.

## 6. Conclusions

A major limitation of the current MICP literature is the lack of standardized reporting. Studies differ in bacterial strain, cell concentration, urea/Ca ratio, pH, temperature, curing time, and soil type, which makes direct comparison difficult. Future work should report a minimum set of parameters to allow meta-analysis. In addition, results obtained in pure cultures or homogeneous sands cannot be directly transferred to heterogeneous field soils, where spatial variability, groundwater flow, and native microbial communities may alter treatment efficiency.

MICP has emerged as a transformative sustainable technology that bridges microbiology, materials science and environmental engineering. This review provides an overview of the mechanisms, influencing factors, and various applications of MICP, emphasizing its potential to address global challenges such as soil erosion, heavy metal pollution, carbon sequestration, and eco-friendly construction. The urease and CA pathways dominate the MICP process and each has unique advantages. Urease-driven MICP allows for rapid carbonate precipitation under controlled conditions, while the CA-mediated pathway excels in CO_2_ sequestration and low urea environments. However, their efficiency is highly dependent on microbial strain selection, environmental parameters (pH, temperature, substrate ratio) and local geochemical conditions.

For the time being, there is a paucity of research on MICP in low-temperature environments, which is an important direction for its future research with a view to expanding its application areas. In addition, with the development of artificial intelligence, the combination of synthetic biology and artificial intelligence may be an emerging research direction for MICP in the future. Synthetic biology and artificial intelligence are promising but currently speculative directions. Genetic engineering has been used to overexpress urease or CA in a few model strains, but it is not yet known whether these strains can survive and function in field soils. Machine learning has been proposed to predict precipitation rates and optimize substrate ratios, but published models are trained on small laboratory datasets and have not been validated across soil types or scales. Before these approaches can be recommended, three questions must be answered: (i) Can engineered strains compete with native microorganisms? (ii) Can models extrapolate beyond their training conditions? (iii) What are the regulatory and biosafety implications? Until then, AI and synthetic biology should be regarded as long-term research directions rather than near-term solutions. In summary, MICP represents a paradigm shift towards sustainable solutions based on biomineralization. Interdisciplinary collaboration across molecular biology, materials engineering and geotechnical engineering is essential to overcome current bottlenecks and unlock its full potential in the real world. Future research should prioritize field trials, life cycle assessments, and regulatory frameworks to ensure safe and scalable deployment of MICP technologies.

## Figures and Tables

**Figure 1:**
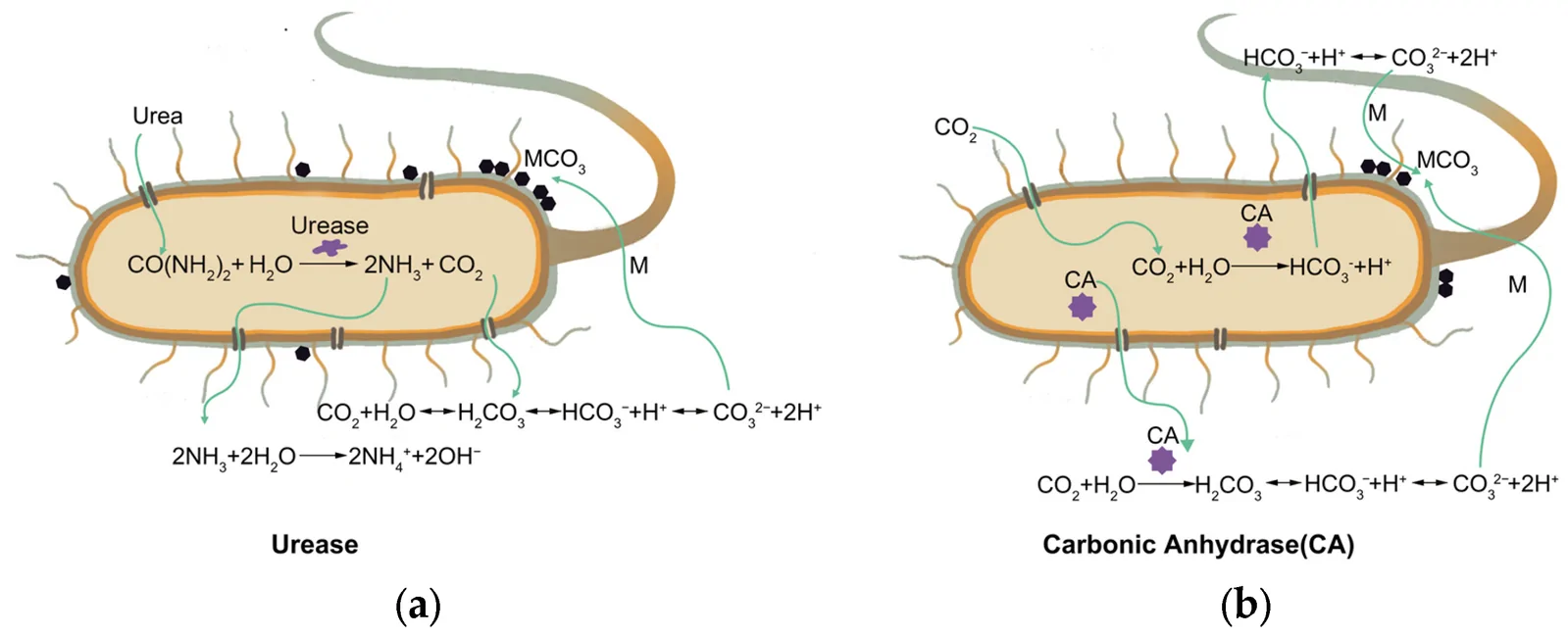
Precipitation of metal cations through MICP. M denotes divalent metal cations (e.g., Ca^2+^, Mg^2+^, Sr^2+^, Ba^2+^, Pb^2+^, Cd^2+^, Cu^2+^, Zn^2+^), which react with carbonate ions to form carbonate precipitates. (**a**) Urease; (**b**) CA.

**Figure 2:**
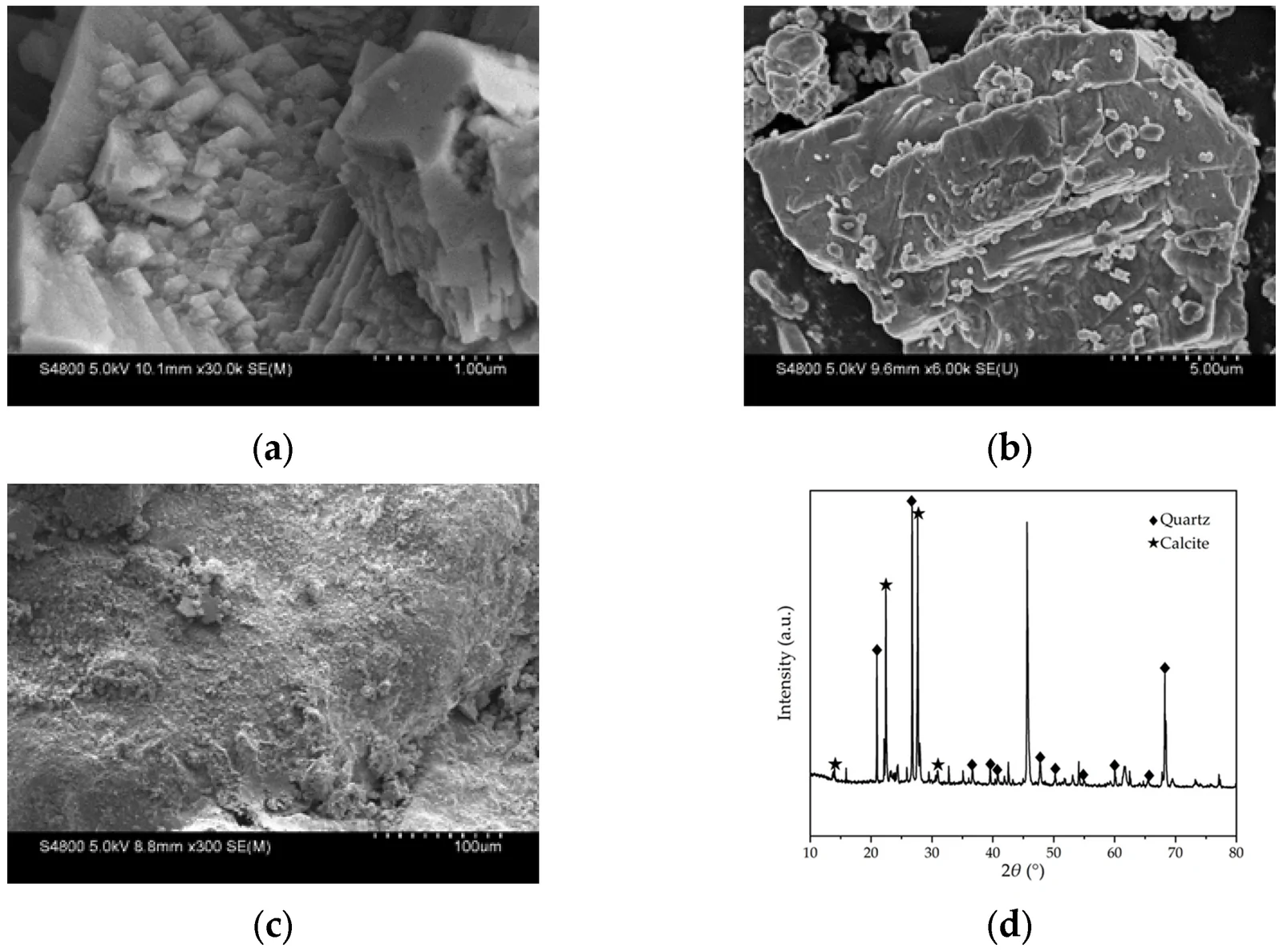
Representative SEM images and XRD pattern of calcium carbonate formed after MICP. (**a**–**c**) SEM images showing typical morphologies. (**d**) XRD pattern with characteristic peaks indexed to calcite. SEM images were obtained in this study. The XRD pattern is reproduced with permission from [14]. Copyright 2024, MDPI.

**Table 1:** A summary of bacteria that entrap or remove heavy metals.

Microorganisms	Heavy Metal Studied	Growth Conditions	Reference
*Acinetobacter* *calcoaceticus*	Zn, Cd	pH 5.0–9.0, C/N 0.5–2.0, Ca^2+^ 10.0–90.0 mg/L, NH_4_^+^-N 5.0–8.0 mg/L	[71]
*Alcaligenes faecalis*	Cd	pH 5.11	[72]
*Bacillus pumilus*	Cr, Cu, Zn	broth media, 37 °C, pH 7.0	[73]
*Bacillus subtilis*	Cr, Cu, Zn	broth media, 37 °C, pH 7.0	[73]
*Bacillus* sp.	Sr	M9 medium, Marine broth 2216	[74,75]
*Bacillus thuringiensis*	Cd	30 °C, 20 and 40 mg/kg Cd^2+^, NB and NA medium	[41,76]
*Brevundimonas diminuta*	Zn, Cd	pH 7.0, LB medium	[77]
*Brucella intermedia*	Cu, Pb, Zn	sterile agar media, 37 °C	[78]
*Citrobacter freundii*	Hg	600 μg/mL, NB medium	[41,79]
*Enterobacter cloacae*	Pb	YA broth and BPU broth	[80]
*Enterobacter hormaechei*	Cd	NB medium, 30 °C	[81]
*Gloeomargarita* *lithophora*	Ra, Sr	BG-11 medium	[82]
*Kocuria flava*	Cu	pH 6.0–9.0, 25–45 °C, Cu^2+^ 100.0–1000.0 mg/L, NB medium	[83]
*Halomonas* sp.	Sr	pH 7.5, 30 °C, Sr^2+^ 100.0 mg/L, NB medium	[84]
*Lysinibacillus capsici*	Cu, Pb	pH 5.16, 30 °C, 0.1–1.6 mmol/L	[85]
*Lysinibacillus fusiformis*	Cd, Cu, Ni, Pb	pH 7.0, 30 °C, NB medium	[55,86,87]
*Lysinibacillus sphaericus*	Th, U, Cu, Pb, Zn, Cd	pH 7.2, 30 °C, LB medium	[31,88,89]
*Oceanobacillus* *indicireducens*	Cr, Cu, Zn	broth media, 37 °C, pH 7.0	[73]
*Serratia marcescens*	Pb, Cd	30 °C, Pb^2+^ 50.0–250.0 mg/L, BPU liquid medium; 30 °C, Cd ^2+^ 100.0 mg/L, LB medium	[90,91]
*Sporosarcina* *ginsengisoli*	As	pH 8.0, 30 °C, As ^2+^ 100.0 mg/L, NB medium	[64]
*Sporosarcina globispora*	Cu, Pb, Co, Zn, Ni, Cd	NH4-YE, 0.5 M urea	[92]
*Sporosarcina koreensis*	Cu, Pb, Co, Zn, Ni, Cd	NH4-YE, 0.5 M urea	[92]
*Sporosarcina pasteurii/Bacillus pasteurii*	Cu, Pb, Co, Zn, Ni, Cd, Cr, Mn, Sr, Eu, U	pH 8.0, 30 °C, 24 mg/L NiCl_2_·6H_2_O, YE medium	[5,54,56,59,62,92,93,94,95,96,97,98,99,100,101,102,103,104,105,106,107,108,109]
*Sporosarcina saromensis*	Cr	30 °C, Cr^2+^ 200.0–500.0 mg/L, DSMZ Medium 1081	[7]
*Sporosarcina ureilytica*	Cd, Pb, Zn	pH 7.55, NB medium	[27,96,106]
*Staphylococcus succinus*	As, Mn, Cu, Ba	pH 7.38, AP1 medium	[110]
*Stenotrophomonas* *rhizophila*	Zn, Pb, Cd	28 °C, NB medium	[111]
*Terrabacter tumescens*	Cu, Pb, Co, Zn, Ni, Cd	NH4-YE, 0.5 M urea	[91]
*Variovorax* *boronicumulans*	Zn, Pb, Cd	28 °C, NB medium	[111]

**Table 2:** A summary of experiments (exp) and applications (app) that restore and conserve building materials.

Microorganisms	Study Object	Co-Adjuvants	Reference
*Bacillus cereus*	cracks in concrete (exp)	zeolite, sodium alginate	[114,115]
*Bacillus cohnii*	the concrete cracks (exp)	carbon sources, nitrogen sources, Mn^2+^	[116]
*Bacillus flexus*	cracks in concrete (exp)	OPC cement	[117]
*Bacillus halodurans*	cracks in concrete (exp)	engineered cementitious composites (ECC)	[118]
*Bacillus mucilaginous*	cracks in concrete (app) (exp)	low-alkali sulfo-aluminate cement (SC), calcium nitrate	[119,120]
*Bacillus sphaericus*	cracks in concrete (exp)	mortar mixtures	[42]
*Bacillus subtilis*	cracks in concrete (exp)	mortar	[121]
*Bacillus thuringiensis*	cracks in concrete (exp)	pH9 buffer	[122]
*Lysinibacillus* *boronitolerans*	cracks in concrete (exp)	Mn^2+^, Fe^2+^, Ca^2+^	[123]
*Lysinibacillus fusiformis*	cracks in concrete (exp)	Mn^2+^, Fe^2+^, Ca^2+^	[124]
*Lysinibacillus* *xylanilyticus*	cracks in concrete (exp)	Mn^2+^, Fe^2+^, Ca^2+^	[124]
*Sporosarcina pasteurii/Bacillus pasteurii*	fracture zone (app) geologic porous media (app) fractured rock (app) cracks in concrete (exp) fractured subsurface rock (app) fractured sandstone(app) fractured rock (exp) fractures shale formations (exp) fracture (app)	mortar mixtures, RCA, lightweight aggregates, basalt fibers, polyester fibers, glass fibers, steel fibers	[42,112,125,126,127,128,129,130,131,132,133,134,135,136,137]

**Table 3:** A summary of bacteria of soil reinforcement and desert management.

Microorganisms	Study Object	Co-Adjuvants	Reference
*Bacillus* *amyloliquefaciens*	sand	calcium salts of formate and acetate	[141]
*Bacillus megaterium*	natural soil, modified soil and manufactured sand	not reported	[142]
*Bacillus subtilis*	sand, sandy soil, silica sand	calcium salts of formate and acetate, silty soil, magnesium chloride	[141,143,144]
*Bhargavaea beijingens*	river sand	not reported	[145]
*Micrococcus* *yunnanensis*	sand	fibers	[57]
*Sporosarcina pasteurii/Bacillus pasteurii*	loess, natural sand, clayey soil, silty soil, saline soil, desert sand, soil, sand, aeolian sand, silty clay, silt soil, field soil, standard sand, white pebble, clayey sand, root–soil composite, loess soil, tailings sand, reclaimed sand, calcareous sand, quartz sand, rammed earth	plant straw, hydroxypropyl methylcellulose, activated carbon, basalt fiber, glutinous rice paste, lignin, polymer	[12,13,142,146,147,148,149,150,151,152,153,154,155,156,157,158,159,160,161]

**Table 4:** A summary of bacteria of CO_2_ sequestration and carbon capture.

Microorganisms	Study Object	Cultivation Conditions	Reference
*Bacillus subtilis*	carbon capture	3 g/L nutrient broth with 20 g/L urea	[29]
*Escherichia coli*	carbon sequestration	LB medium, 500 mL flask, 37 °C	[166,167]
*Nannochloropsis* *oceanica*	carbon capture systems	F/2 Medium	[168]
*Sporosarcina pasteurii/* *Bacillus pasteurii*	carbon capture, carbon sequestration	Modified Nutrient Broth medium	[169]

**Table 5:** A summary of bacteria of new biomaterials and technologies.

Microorganisms	Study Object	Reference
*Bacillus halodurans*	Genetically Modified Bacteria	[118]
*Bacillus licheniformis*	bio self-healing dental composite	[172]
*Bacillus megaterium*	bio self-healing dental composite	[172]
*Bacillus sphaericus*	bio self-healing dental composite	[172]
*Bifidobacterium longum*	bio self-healing dental composite	[172]
*Lactobacillus reuteri*	bio self-healing dental composite	[172]
*Sporosarcina pasteurii/* *Bacillus pasteurii*	3D printing, Micromodels, Biocement Bricks, Biocement, bio-tiles	[17,164,173,174,175,176,177,178,179]
*Streptococcus salivarius*	bio self-healing dental composite	[172]
*Synechococcus* sp. PCC 7002	Living building materials	[170]

## Data Availability

No new data were created or analyzed in this study. Data sharing is not applicable to this article.
